# THE CRITICAL VIEW OF SAFETY PREVENTS THE APPEARANCE OF BILIARY INJURIES? ANALYSIS OF A SURVEY

**DOI:** 10.1590/0102-672020180001e1380

**Published:** 2018-07-02

**Authors:** Mariano Eduardo GIMÉNEZ, Eduardo Javier HOUGHTON, Manuel E. ZELEDÓN, Mariano PALERMO, Pablo ACQUAFRESCA, Caetano FINGER, Edgardo SERRA

**Affiliations:** 1 University of Buenos Aires, President DAICIM Foundation; 2 Mini-Invasive Surgery, Hospital Bernardino Rivadavia, University of Buenos Aires, Staff DAICIM Foundation; 3 University of Costa Rica; 4 Staff DAICIM Foundation and University of Buenos Aires; 5 Staff DAICIM Foundation, Buenos Aires, Argentina

**Keywords:** Laparoscopic cholecystectomy, Biliary duct injury, Critical view of safety., Colecistectomia laparoscópica, Lesão do duto biliar, Visão crítica da segurança.

## Abstract

***Background*:**

The risk of bile duct injury (BDI) during cholecystectomy remains a concern, despite efforts proposed for increasing safety. The Critical View of Safety (CVS) has been adopted promoting to reduce its risk.

***Aim*:**

To perform a survey to assess the awareness of the CVS, estimating the proportion of surgeons that correctly identified its elements and its relationship with BDI.

***Methods*:**

An anonymous online survey was sent to 2096 surgeons inquiring on their common practices during cholecystectomy and their knowledge of the CVS.

***Results*:**

A total of 446 surgeons responded the survey (21%). The percentage of surgeons that correctly identified the elements of CVS was 21.8% and 24.8% among surgeons claiming to know the CVS. The percentage of surgeons that reported BDI was higher among those that incorrectly identified the elements of the CVS (p=0.03). In the multivariate analysis, career length was the most significant factor related to BDI (p=0.002).

***Conclusions*:**

The percentage of surgeons that correctly identified the Critical View of Safety was low, even among those who claimed to know the CVS. The percentage of surgeons that reported BDI was higher among those that incorrectly identified the elements of the CVS.

## RESUMO

***Racional*:**

O risco de lesão do ducto biliar (BDI) durante a colecistectomia continua a ser preocupante, apesar dos esforços propostos para aumentar a segurança. A Visão Crítica da Segurança (CVS) foi adotada e proposta para redução de seu risco.

***Objetivo*:**

Realizar levantamento para avaliar a conscientização da CVS, e estimando a proporção de cirurgiões que identificaram corretamente seus elementos e sua relação com BDI.

***Métodos*:**

Pesquisa online anônima foi enviada para 2096 cirurgiões perguntando sobre suas práticas comuns durante a colecistectomia e seu conhecimento da CVS.

***Resultados*:**

Um total de 446 cirurgiões responderam a pesquisa (21%). A porcentagem que identificou corretamente os elementos da CVS foi de 21,8% e 24,8% entre os cirurgiões que afirmam conhecê-la. A porcentagem dos que relataram BDI foi maior entre os que incorretamente identificaram os elementos da CVS (p=0,03). Na análise multivariada, o tempo na carreira foi o fator mais significativo relacionado à BDI (p=0,002).

***Conclusões*:**

A porcentagem de cirurgiões que identificaram corretamente a CVS foi baixa, mesmo entre aqueles que alegaram conhecê-la. A porcentagem de cirurgiões que relataram BDI foi maior entre aqueles que incorretamente identificaram os elementos da CVS.

## INTRODUCTION

Iaparoscopic cholecystectomy (LC) is the gold standard for management of gallstones^1^ ^1^. However, the risk of bile duct injury (BDI) remains a significant concern^10^, as LC continues to have a higher BDI rate than its open counterpart, despite many efforts proposed for increasing safety^12^ ^,^ ^13^ ^,^ ^14^ ^,^ ^15^ ^,^ ^16^ ^,^ ^17^ ^,^ ^18^ ^,^ ^19^ ^,^ ^20^ ^,^ ^21^ ^,^ ^22^ ^,^ ^23^ ^,^ ^24^ ^,^ ^25^ ^,^ ^26^. 

The Critical View of Safety (CVS) proposed by Strasberg^22^, is a technique for identification of the critical elements of the Calot triangle during LC. This technique has been adopted in several teaching programs and with the proposition to reduce the risk of BDI^6^ ^,^ ^8^ ^,^ ^7^ ^,^ ^9^ ^,^ ^11^ ^,^ ^12^ ^,^ ^13^ ^,^ ^14^ ^,^ ^15^ ^,^ ^16^ ^,^ ^17^ ^,^ ^18^ ^,^ ^19^ ^,^ ^20^ ^,^ ^21^ ^,^ ^22^ ^,^ ^23^ ^,^ ^24^ ^,^ ^25^ ^,^ ^26^ ^,^ ^27^. However, despite its application, BDI rates have not decreased even in centers where it is routinely used^14^ ^,^ ^19^; this phenomenon has been analyzed in several studies^15^ ^,^ ^20^ ^,^ ^21^ ^,^ ^22^ ^,^ ^23^. The use of CVS, however, is associated with lower BDI rates^1^ ^,^ ^2^ ^,^ ^3^ ^,^ ^4^ ^,^ ^5^ ^,^ ^6^ ^,^ ^7^ ^,^ ^8^ ^,^ ^9^ ^,^ ^10^ ^,^ ^11^ ^,^ ^12^ ^,^ ^13^ ^,^ ^14^ ^,^ ^15^ ^,^ ^16^ ^,^ ^17^ ^,^ ^18^ ^,^ ^19^ ^,^ ^20^ ^,^ ^21^ ^,^ ^22^ ^,^ ^23^ ^,^ ^24^ ^,^ ^25^ ^,^ ^26^ ^,^ ^27^ ^,^ ^28^ ^,^ ^29^, therefore the possibility of incorrect application of CVS should be identified promptly if there is hope to benefit from its application. 

Assessing safe LC is an arduous task; however, it remains a priority for many organizations. Examples of these efforts include the launch of the Safe Cholecystectomy Task Force by the Society of American Gastrointestinal Endoscopic Surgeons in 2014^19^ and the Dutch Health Care Inspectorate making CVS mandatory in the Netherlands in 2009 ^5^. 

As an effort to participate in the global endeavor for increasing LC safety, we performed a survey of surgeons in Latin America. Our main objectives included assessing the awareness of CVS, estimating the proportion of surgeons that correctly identified its elements, and its relationship with BDI. Also, were analyzed the relationship among other factors such as career length, intraoperative cholangiography (IOC) and workplace. 

## METHODS

This is a prospective, observational, comparative and transverse study. It was conducted after approval from the Investigation Ethics Committee of the Bernardino Rivadavia Hospital (No.DC-2017-296-HBR).

In June of 2017, a total of 2340 email addresses of surgeons were selected from the database of the DAICIM Foundation (Buenos Aires, Argentina) as recipients for an anonymous online survey. A form was sent by email (using Google Forms by Google). The survey was closed once the estimated sample size was obtained. Surgeons working in Latin America were included and forms that were incompletely filled, excluded. The main outcome was the percentage of surgeons that reported BDI, comparing with the independent variable “correctly identified elements of CVS”, with control of the variable “career length”.

### Statistical analysis

All statistical analyses were performed using SPSS 11.5 y VCCstat^2^. When necessary the standard deviations and confidence intervals of 95% (CI95) were estimated, and the following statistical significance tests were applied: Students T-test, ANOVA, Chi-squared, and Fishers test. A p-value of <0.05 was considered as significant statistically. For the multivariate analysis, a binary logistic regression was performed with an alfa entry level=0.05 and an exit alfa of =0.10. 

## RESULTS

A total of 446/2096 (Table 1, Figure 2) surgeons answered the questions (response rate of 21.2%); 244 contacts were badly addressed and one survey was discarded due to incomplete information; therefore, 445 surveys were qualified. 

**FIGURE 1:**
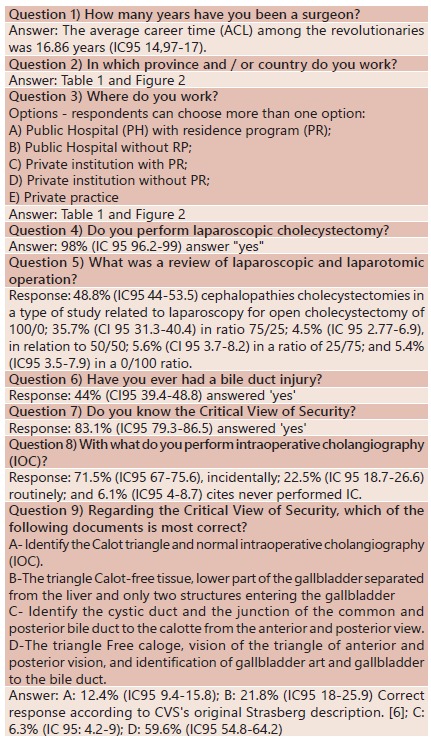
Online questions and answers done to the surgeons

Questions and answers done to the surgeons are in Figure 1.

Regarding the main objectives, 78,3% (CI95 74-82) answered incorrectly the question about the correct definition of the CVS, consequently only 21,8% (CI95 18-25,9) correctly identified the CVS criteria. Among those who claimed to know the CVS, only 24.8% (CI95 20.6-29.6) answered it correctly. Among the group that claimed to know the CVS but incorrectly identified its elements, 46.8% (CI95 41.5-52.2) reported having BDI vs. 34% (CI95 24.7-44.3) among those that claimed to know the CVS and correctly identified its elements. This difference was statistically significant (ChiYates p=0.03, Table 2). 

Of the 92 surgeons that correctly identified the elements of the CVS, 33 reported BDI (35.9%) (CI95 26.1-46.5), and among the 348 that incorrectly identified them, 163 reported BDI (48.5%)(CI95 42.5-54.6) (Chi Yates p=0,0457). Five surgeons admitted that they did not know the CVS; however, they correctly identified its elements (all five did not report BDI); as their correct answers were random, they were excluded from the previous analysis. 

In regard to analyzing a possible relation in the average career length with the correct identification of the elements of the CVS, the results showed that the average career length of respondents was 16.86 years (CI95 14.97-17). However, among surgeons that correctly identified the elements of the CVS, the average was 12.58 (CI95 10.18-13.81), whereas it was 18.06 (CI95 16.73-19.26) among those that incorrectly identified the elements of the CVS (T test p=0.0005). 

**TABLE 1:** Total number of participants according to the country they presently worked in

Country	Total number of participants
Argentina	326
Uruguay	32
Peru	21
Ecuador	5
Guatemala	3
Mexico	12
Bolivia	22
Venezuela	5
Paraguay	9
Colombia	1
Cuba	1
Brazil	2
Costa Rica	3
Chile	3

**FIGURE 2:**
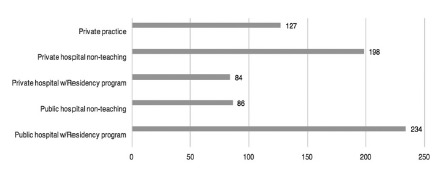
Workplace of respondents

**TABLE 2:** BDI among surgeons that claimed to know the CVS, but incorrectly identified the elements vs. surgeons that claimed to know the CVS, but correctly identified the elements

	Reported BDI	No BDI	Total
Correctly identified CVS elements	33	64	97
Incorrectly identified CVS elements	163	185	348
Total	196	249	445

The average career length among surgeons that reported BDI was 19.32 (SD 12.36 N: 196. CI95 17.3-20.7) and 15.04 (SD 11.34 N: 249 CI95 13.62-16.37) among those who did not (T test p=0.00028). Therefore, as both longer “career length” and incorrect “identification of the elements of CVS” were statistically associated with “reported BDI”, a logistic regression multivariate analysis was necessary to determine which one was more relevant. 

This analysis was performed using as independent variables: “identification of the elements of CVS” and “career length”, to predict the appearance of the event “BDI”. As a result, “career length” was the most significant factor related to a higher percentage of surgeons reporting BDI (p=0.0002). The threshold was found between 15 and 19 years, and above a set value of 16 years, the risk of reporting BDI is 1.7 times increased (OR 1.7 CI95 1.14-2.44, Table 3)

**TABLE 3:** Reported BDI by career length

	Reported BDI	Non reported BDI	Total
Above 16 Years	102	98	200
Bellow or equal to 16 years	94	151	245
Total	196	249	445

On the subject of the use of IOC and surgeons reporting BDI, of those routinely performing IOC, 48% (CI 95 37.87-58.24) reported BDI; amid those that performed IOC incidentally, 43.4% (CI95 37.8-49) and finally, 37% (CI95 19.34-57.68) of surgeons never performing IOC reported BDI. The difference among these three groups was not statistically significant (p=0.54 Squared Chi,Table 4). 

**TABLE 4:** Reported BDI by used of IOC

IOC	With BDI	Without BDI	TOTAL
Never	10	17	27
Incidentally	138	180	318
Routinely	48	52	100
TOTAL	196	249	445

We analyzed if a larger number of surgeons reported BDI in non-teaching centers vs. those in Surgical Residency Program (SRP) centers. The results were as follows: 289 respondents worked in SRP centers, of these, 124 reported BDI (42.9% CI95 37.1-48.8). Among surgeons in non-teaching centers, 72 (46.15% CI95 38.1-54.3) reported BDI out of a total of 156; this difference was not statistically significant (p=0.57 Chi-Yates test). 

Because of the absence of a statistical difference in the previous analysis, the career length between non-teaching centers and SRP centers was also analyzed. In non-teaching centers, the average career length was 20.22 (SD: 12.08) and in SRP centers, the average was 15.08 (SD=11.56, p=0.000001, t Student). 

To reveal if surgeons that correctly identified the CVS were associated alongside SRP centers, was performed the following analysis: the number of surgeons in SRP centers that identified the CVS correctly was 74 (25.6% CI95 20.6-31), while 215 answered incorrectly. In non-teaching centers, 23 (14.7% CI95 9.56-21.3) answered correctly, while 133 answered incorrectly; this difference was statistically significant (p=0,004 Fishers test, p=0.01 Chi-Yates test).

When analyzing if the percentage of surgeons that routinely and incidentally perform IOC was higher in SRP centers, the results (Table 5), showed that there was a statistically significant difference favoring those working with residents when compared to those in non-teaching centers (Chi Yates p=0.003). 

**TABLE 5:** Use of IOC by workplace

	Never IOC	Incidentally IOC	Routinely IOC	Total
SRP Centers	10 (3.46% IC95 1.76-6.5)	206 (71.3% IC95 65.3-76.3)	73 (25.2% IC95 20.4-30)	289
Non teaching centers	17 (10.9% IC95 6.6-17.1)	112 (71.8% IC95 64-78.5)	27 (17.3% IC95 11.9-24.3)	156

## DISCUSSION

Bile duct injury during LC is a distressing event that can significantly alter a patient’s life. Strasberg’s CVS has emerged as a useful tool for improving safety^5^ ^,^ ^6^ ^,^ ^27^. However, some reports highlighted that despite of its use, the incidence of BDI has not necessarily decreased^15^ ^,^ ^20^ ^,^ ^21^ ^,^ ^22^ ^,^ ^23^. Several studies have suggested education of CVS, strict video or photographic documentation of it during surgeries, and even confronting surgeons with their low results, as methods to increase the impact of CVS use^4^ ^,^ ^15^ ^,^ ^20^. 

A similar large-scale, multinational survey, by Hibi et al.^1^ ^3^, found that surgeons’ perceptions during LC are workplace-dependent, and some common indices are collectively inapplicable in multicenter, international trials; this overlaps with safety measures such as the use of CVS. In the same way, an evaluation of LC protocols of Dutch hospitals in 2008 by Wauben L. et al.^28^ found that even in this setting, protocols differed widely and the sections relating to the CVS, presented omissions such as: many protocols not mentioning the terms ‘Calot´s triangle dissection’ or not describing its complete dissection. These studies suggest that awareness and the correct application of the CVS may be dissimilar. 

The present survey found that a surprising 78% of respondents did not recognize correctly the elements of the CVS. But more concerning, was the finding that out of the group of surgeons that claimed to know the CVS, 75% were mistaken. Because most studies on the subject of CVS and its use, have not clearly stated that the surgeons performing the LC were certified as to knowing the elements of the CVS^6^ ^,^ ^1^ ^5^ ^,1^ ^6^ ^,^ ^2^ ^5^ ^,^ ^26^- even though the premise of surgeons confirming the obtainment of CVS presupposes their knowledge on the subject - our findings suggest that confirmation of the fact, may be necessary and that this observation could be an explanation as to why CVS has not had the impact it should have. 

Our results also found that surgeons with a shorter surgical career were more aware of the CVS; this could support that the recent contact with a training program might be associated with awareness of the CVS. Similarly, the results showed a significant association of surgeons working in SRP centers with knowledge of the CVS and a shorter surgical career. These, results support the notion that CVS is a relatively “young” technique that is commonly found among young surgeons in academic settings. This situation proposes prioritizing the dissemination of CVS among older surgeons and those working in non-teaching centers.

Other findings of this study included that a greater knowledge of the CVS and a shorter surgical career were both associated with surgeons that did not report BDI. However, in a multivariate analysis, career length was the more significant factor related with the appearance of BDI (p=0.0002), including the risk of BDI increasing almost two-fold (OR 1.7 CI95 1.14-2.44) after 16 years. This result suggests that, at present, a longer surgical career is more of a risk factor for the appearance of BDI, than ignorance of the CVS. 

With these associations, it would follow that surgeons working in SRP centers (younger surgeons, more aware of the CVS) would logically have lower BDI reports; however, this was not the case. In a comparison of the reports of BDI between, centers with SRP and non-teaching centers, the response rate for BDI was not statistically significant (p=0.57 Chi-Yates test). Several possible explanations could be responsible for this ‘equalization’ between BDI rates among SRP vs. non-teaching centers. LC with higher degrees of difficulty in SRP centers, with a corresponding selection of “easier” cases in non-teaching centers, would likely be the most obvious factor that could simultaneously increase BDI in the former while decreasing it in the latter. Furthermore, more experience in “older” surgeons in non-teaching centers, and incorrect CVS application by “younger” surgeons in SRP centers, could also play a part in this finding.

Our results also found, as mentioned in previous reports, that IOC was not associated with lower reports of BDI^8^ ^,^ ^9^ ^,^ ^10^ ^,^ ^11^ ^,^ ^12^ ^,^ ^13^ ^,^ ^14^ ^,^ ^15^ ^,^ ^16^ ^,^ ^17^. However, unlike other authors that have suggested that IOC is becoming an endangered technique^6^, over 90% of respondents to the survey admitted to performing IOC at some point, therefore it seems that reports promoting the benefits of it^2,3,4,5,6,7,8,9,10,11,12,13,14,15,16,17,18,19,20,21,22,23,24^ continue to promote IOC as a risk-reducing technique and might explain why it seems to still be very alive among surgeons in the area.

Our study has some limitations. Twenty percent of response rate could be pointed out very low; however, according to Sheehan^22^ the response rates to email surveys have been decreasing over time and by the beginning of the millennium, they oscillated by 20%. Our response rates was within that range^21^. In the survey, we did not ask the surgeons the exact number of BDI that they had incurred in their careers. Therefore, our analysis could not differentiate between surgeon’s experience and the accumulative effect of time in relation to BDI. Secondly, we described that the percentage of surgeons reporting BDI was lower in the group that correctly identified the elements of CVS. However, correctly identifying them is not the same as using correctly and routinely in practice, and assuming that, could be a potential bias of our study.

Finally, our conclusions include that the percentage of surgeons that correctly identified the elements of CVS was much lower than expected (21.8%) even among those who claimed to know the CVS. Therefore, this aspect should be noted in future investigations and in educational programs. Also, the percentage of surgeons that reported BDI was higher among those that incorrectly identified the elements of the CVS; however .a longer career length was the most significant factor related to BDI.

## CONCLUSIONS

The percentage of surgeons that correctly identified the Critical View of Safety was low, even among those who claimed to know the CVS. The percentage of surgeons that reported BDI was higher among those that incorrectly identified the elements of the CVS.

## References

[1] Abbasoglu O (2016). Prevention and acute management of biliary injuries during
laparoscopic cholecystectomy Expert consensus statement. Turk J Surg.

[2] Alvarez FA (2014). Impact of routine intraoperative cholangiography during
laparoscopic cholecystectomy on bile duct injury. Br J Surg.

[3] Avgerinos C (2009). One thousand laparoscopic cholecystectomies in a single surgical
unit using the "critical view of safety" technique. J Gastrointest Surg.

[4] Buddingh KT (2012). Documenting correct assessment of biliary anatomy during
laparoscopic cholecystectomy. SurgEndosc.

[5] Buddingh KT (2011). Intra- operative assessment of biliary anatomy for prevention of
bile duct injury a review of current and future patient safety
interventions. SurgEndosc.

[6] Buddingh KT (2011). Safety measures during cholecystectomy results of a nationwide
survey. World J Surg.

[7] De Mestral C (2014). Comparative operative outcomes of early and delayed
cholecystectomy for acute cholecystitis a population-based propensity score
analysis. Ann Surg.

[8] Debru E (2005). Does routine cholangiography prevent bile duct
transection. SurgEndosc.

[9] Ding GQ (2015). Is intraoperative cholangiography necessary during laparoscopic
cholecystectomy for cholelithiasis. World J Gastroenterol.

[10] Giménez ME, Palermo M, Houghton E, Acquafresca P, Finger C, Verde JM, Cúneo JC (2016). Biodegradable biliary stents: a new approach for the management
of hepaticojejunostomy strictures following bile duct injury. Prospective study. Arq Bras Cir Dig.

[11] Gollan J (1992). Gallstones and laparoscopic cholecystectomy, NIH
Consens. Statement.

[12] Henneman D (2013). Laparoscopic partial cholecystectomy for the difficult
gallbladder a systematic review. SurgEndosc.

[13] Hibi T (2017). The "right" way is not always popular comparison of surgeons'
perceptions during laparoscopic cholecystectomy for acute cholecystitis
among experts from Japan, Korea and Taiwan. Hepatobiliary Pancreat Sci.

[14] Khan MH (2007). Frequency of biliary complications after laparoscopic
cholecystectomy detected by ERCP experience at a large tertiary referral
center. GastrointestEndosc.

[15] Nijssen MA (2015). Complication after laparoscopic cholecystectomy a video
evaluation study of whether the critical view of safety was
reached. World J Surg.

[16] Nijssen MA. (2016). Improving Critical View of Safety in Laparoscopic Cholecystectomy
by Teaching Interventions. J Surg Educ.

[17] Nuzzo G (2005). Bile duct injury during laparoscopic cholecystectomy results of
an Italian national survey on 56 591 cholecystectomies. Arch Surg.

[18] Pekolj J (2013). Intraoperative management and repair of bile duct injuries
sustained during 10,123 laparoscopic cholecystectomies in a high-volume
referral center. J Am Coll Surg.

[19] Pucher PH (2015). SAGES expert Delphi consensus critical factors for safe surgical
practice in laparoscopic cholecystectomy. SurgEndosc.

[20] Sanford DE, Strasberg SM (2014). A simple effective method for generation of a permanent record of
the critical view of safety during laparoscopic cholecystectomy by
intraoperative doublet photography. J Am Coll Surg.

[21] Sheehan K. B (2001). "E-mail survey response rates: A review". Journal of Computer-Mediated Communication.

[22] Strasberg SM, Hertl M, Soper NJ (1995). An analysis of the problem of biliary injury during laparoscopic
cholecystectomy. J Am Coll Surg.

[23] Tornqvist B (2012). Effect of intended intraoperative cholangiography and early
detection of bile duct injury on survival after cholecystectomy popula- tion
based cohort study. BMJ.

[24] Tornqvist B (2015). Selective intraoperative cholangiography and risk of bile duct
injury during cholecystectomy. Br J Surg.

[25] Tsalis K (2015). Open-access technique and &raquo;,» &reg;,® &sect;,§
&shy;,­ &sup1;,¹ &sup2;,² &sup3;,³ &szlig;,ß
&THORN;,Þ &thorn;,þ &times;,× &Uacute;,Ú &uacute;,ú
&Ucirc;,Û &ucirc;,û &Ugrave;,Ù &ugrave;,ù &uml;,¨
&Uuml;,Ü &uuml;,ü &Yacute;,Ý &yacute;,ý &yen;,¥
&yuml;,ÿ &para;,¶ critical view of safety &raquo;,» &reg;,®
&sect;,§ &shy;,­ &sup1;,¹ &sup2;,² &sup3;,³
&szlig;,ß &THORN;,Þ &thorn;,þ &times;,× &Uacute;,Ú
&uacute;,ú &Ucirc;,Û &ucirc;,û &Ugrave;,Ù &ugrave;,ù
&uml;,¨ &Uuml;,Ü &uuml;,ü &Yacute;,Ý &yacute;,ý
&yen;,¥ &yuml;,ÿ &para;,¶ as the safest way to perform
laparoscopic cholecystectomy. SurgLaparoscEndoscPercutan Tech.

[26] Vettoretto N, Saronni C, Harbi A (2011). Critical view of safety during laparoscopic
cholecystectomy. JSLS.

[27] Vollmer CM, Callery MP (2007). Biliary injury following laparoscopic cholecystectomy why still a
problem?. Gastroenterology.

[28] Wauben L (2008). Evaluation of Protocol Uniformity Concerning Laparoscopic
Cholecystectomy in The Netherlands. World J Surg.

[29] Yegiyants S (2008). Operative strategy can reduce the incidence of major bile duct
injury in laparoscopic cholecystectomy. Am Surg.

